# Turning over New Leaves

**Published:** 1892-10-15

**Authors:** 


### Turning Over New Leaves.

We have received a copy of Hadden's "Pocket Vocabulary of Medical Terms," the second edition of a handy little volume already well-known to many. The book deals with the most frequently used medical terms, such as may be used within the hearing of nurses every day, and to them it will be found especially useful as a first book of the kind, leading up to works of a more strictly scientific and comprehensive character. The new edition contains one hundred additional words, all useful and well selected. The "Vocabulary" is printed clearly on good paper, is handy in form, and moderate in price.

